# The Book World of Medicine and Science

**Published:** 1904-04-30

**Authors:** 


### The Book World of Medicine and Science.

Divine Hygiene. By Alexander Rattray, M.D. (Edinburgh: James Nisbet and Co. Two vols. 32s.)

This work is the outcome of many years' study and of much patient industry, but also, we fear, of some misplaced energy. Each of its two ponderous volumes contains upwards of six hundred pages, and judging from its size, its well-arranged and exhaustive tables, and from the enormous number of references we can form some idea of the industry of its author. The work purports to deal with the "Sanitary Science and Sanitarians of the Sacred Scriptures and Mosaic Code," and after a tedious and somewhat complicated introduction we reach chapters on various hygienic subjects as dealt with in the Bible. These chapters discuss at great length the Hygiene of Health, Personal Hygiene, Food and Drink, Air, Exercise, Cleanliness, Clothing, Heredity, etc. The latter part of the work describes and sets forth the Hygiene of Disease and the Mosaic Code from a modern medical point of view. Some of these chapters commence with the author's own view of the contemporary aspect of the subject, and a great deal of miscellaneous information is given, although its connection with the Mosaic Code is not always apparent. For instance, in the chapter on exercise we read that "in a Boston normal school for teachers, in which the graduates were about equally divided between the sexes, in every one of the ten classes the best gymnast was a woman." Of course Moses is the hero of the book. He was a genius; carefully educated; studied medicine at Heliopolis, or On; and he had ample time to complete his studies during his forty years in the solitudes of Midian, and ended by being a "highly talented physician." This medical education of Moses is one of those particularly fine things which we are treated to more than once, for it appears on page 568 and on page 623. After this we are not much surprised to hear that Moses was a microscopist and a bacteriologist. Dr. Rattray is so little of an evolutionist that he traces leprosy and other diseases to the Fall. But does not this place him very nearly between the horns of a dilemma? For either the microbes of diseases were created in the Garden of Eden or they are products of evolution, unless there were a second creation, of which we have no record at all. Dr. Rattray is apparently an American, though he has a British doctorate. We should like to know what his fellow-citizens think of this work with its cumbrous style, its peculiar punctuation, and its rather Teutonic form of construction of sentence. We read that "while God and His two records are true; God Himself is truth; scientists and divines truth seekers, nor should the noteworthy fact be forgotten, that not a few of the acknowledged leaders of science have been devout men." On page 349 of vol. i. we are told that "Jehovah kept them pretty busy," which though true is not exactly an ideal way of expressing God's activity. It would be ungracious not to note that Dr. Rattray, in addition to his painstaking industry, shows a very sincere and whole-hearted devotion to his undertaking, and that his volumes will form an admirable reference work for anyone who may wish to preach or write upon Mosaic hygiene.

Diseases of the Gall-Bladder and Bile Ducts. By Mayo Robson, F.R.C.S., Vice-President of the Royal College of Surgeons of England, assisted by J. F. Dobson, M.S., F.R.C.S. Third Edition. (London: Baillière, Tindall and Cox. 1904. Pp. 485. 73 Illustrations. Price 15s. net.)

This is the third edition of Mr. Mayo Robson's well-known work on the subject, but as the author states in his preface it may be taken as an almost entirely new book. Whereas the previous edition had only 313 pages this has 485, and the number of the illustrations has been increased from 52 to 73. The volume is a complete monograph on the subject, the only noteworthy omission being a bibliography of the chief literature upon the matters dealt with, in addition to the names of the authors quoted. It is hardly necessary to traverse the contents of the book as they are so well known by the previous editions, but it may be stated that the following parts of the work are quite new: a chapter on physiological considerations, another on injuries of the bile passages, an additional paragraph on kinking of the bile-ducts. The chapter on gall-stones and their treatment has been rewritten, and is one of the best in the work. In the appendix is placed a record of no fewer than 539 operations performed by the author in this branch of surgery, and this is a most valuable practical addition to the book, as indicating the experience upon which the statements in the body of the volume have been made. The anatomical considerations give a good account of the chief facts in connection with the liver, gall-bladder, and the biliary passages. In the chapter on physiological considerations much of importance has been introduced, and the author's original observations are of great value. The question of injuries to the gall-bladder and the bile-ducts is ably reviewed and the opinion that these cases, if diagnosed, should be immediately submitted to operation, strongly supported. In inflammatory affections of the same parts, we do not think that sufficient emphasis is laid upon the bacterial origin of the catarrh, even in cases in which no suppuration occurs. The results of inflammation are excellently reviewed, and the chapter is one which cannot fail to excite interest and impart knowledge. The author still considers that obstruction of the intestine from the impaction of a gall-stone is a comparatively rare affection, and he is probably right, but there are possibly cases in which death supervenes without the cause of the obstruction being recognised as that of an impacted gall-stone. He further alludes to the significant fact that very few of the fatal cases following upon laparotomy for such a condition are published, and these again would add to the number, which cannot be inconsiderable. Special attention should be drawn to the admirable *resumé* of the preparation of a patient for an operation upon the biliary passages. There are a few points which would be better altered in any future edition. First, is the spelling of the name of the well-known professor of surgery at Baltimore, whose name appears on some pages rightly spelt, and on others improperly so, and secondly, some of the illustrations of specimens would be greatly improved if re-photographed, and it is unhappy that such was not done before the present edition was published. We refer to such reproductions as those depicted in Figs. 11, 22, and others. With these and a few other minor imperfections corrected, the work is one which must rank high in surgical literature.

Anæsthetics in Surgery. By C. Hamilton Whiteford. (Bristol: J. W. Arrowsmith. Price 1s. 6d. net.)

This brochure is based on a series of 2,000 consecutive inductions of general anæsthesia and reporting the author's experience and his conclusions deduced therefrom. The A.C.E. mixture has been given up by him owing to there being no apparent advantage in it. The use of chloroform is also deprecated owing to dangers of collapse or even loss of life. Ethyl chloride is commented on favourably, and is described as likely in many cases to supersede the use of nitrous oxide for short operations. We think that a perusal of this small pamphlet would repay anyone interested in the giving of anæsthetics.

